# Renal hypertrophy and hyperfiltration is enhanced in early acquired compared with a congenital solitary function kidney model in sheep

**DOI:** 10.1042/CS20243031

**Published:** 2025-03-13

**Authors:** Zoe McArdle, Reetu R. Singh, Sarah L. Walton, Karen M. Moritz, Kate M. Denton, Michiel F. Schreuder

**Affiliations:** 1 Cardiovascular Disease Program, Monash Biomedicine Discovery Institute and Department of Physiology, Monash University, Melbourne, Australia; 2 Child Health Research Centre and School of Biomedical Sciences, The University of Queensland, Brisbane, Queensland, Australia; 3 Department of Paediatric Nephrology, Amalia Children’s Hospital, Radboud University Medical Centre, Nijmegen, The Netherlands

**Keywords:** congenital solitary functioning kidney, early acquired solitary functioning, hyperfiltration, nitric oxide, renal functional reserve

## Abstract

A congenital solitary functioning kidney (C-SFK) or an early acquired SFK (EA-SFK), due to childhood unilateral nephrectomy (UNX), increases the risk of hypertension and kidney disease early in life. Evidence suggests that children with an EA-SFK may have a higher risk of future kidney disease compared with those with a C-SFK, but the precise underlying mechanisms need further investigation. C-SFK was induced by fetal UNX at 100 days gestation (term=150 days) in male sheep fetuses, and a sham procedure was performed. At approximately one month of age, EA-SFK was induced by UNX in male lambs. At eight months of age, total kidney weight was similar in all groups due to marked hypertrophy in the C-SFK and EA-SFK groups. Blood pressure was similar in EA-SFK and sham groups but ~12 mmHg higher in the C-SFK group compared with sham. Compared with the sham group, glomerular filtration rate (GFR) was ~9% less in the EA-SFK group and ~26% less in the C-SFK. GFR was ~23% higher in EA-SFK compared with the C-SFK group. Albuminuria was ~67% higher in C-SFK sheep but similar in the EA-SFK group compared with sham sheep. However, like the C-SFK group, the renal blood flow response to nitric oxide blockade was attenuated in the EA-SFK group compared with sham. In conclusion, long-term studies are needed to determine whether the higher hyperfiltration and disturbed vasodilator balance observed in EA-SFK sheep will cause an accelerated decline in renal function with aging.

## Introduction

Being born with one kidney (congenital solitary functioning kidney [C-SFK]) or having a kidney removed early in life (early acquired SFK [EA-SFK]), via childhood unilateral nephrectomy (UNX), increases the risk of hypertension and chronic kidney disease (CKD) [1–5]. Recently, it was demonstrated that ~75% of C-SFK and ~80% of EA-SFK showed at least one sign of kidney injury by 18 years of age [1]. This is in marked contrast with the loss of a kidney during adulthood, via kidney donation, in which blood pressure and kidney function are generally preserved and most patients live long healthy lives [6,7]. Kidney loss early in life causes a greater degree of compensatory kidney hypertrophy and an increase in glomerular filtration rate (GFR) in the remaining kidney compared with kidney loss in adulthood, which likely contributes to the age-related differences in outcomes observed [8,9].

The timing of neonatal kidney loss may also differentially affect the risk of future decline in kidney function and hypertension. In humans, nephrogenesis is complete before birth (week 36 of gestation) [10]. Studies indicate that a C-SFK has the capacity to prolong the period of nephrogenesis and partially compensate for the reduction in nephron number associated with kidney loss, with total nephron number in the C-SFK closer to ~70–75% of two kidney controls [11–13]. The level of compensatory nephrogenesis may confer protection from future hyperfiltration-mediated kidney injury in individuals with C-SFK [14]. In comparison, in the EA-SFK, UNX occurs after the completion of nephrogenesis when new nephrons no longer form, resulting in a ~50% reduction in nephron number. Therefore, the final reduction in nephron number may be greater in EA-SFK than C-SFK. Approximately 1:2000 children are born with a C-SFK [15]. Pediatric UNX is most common in the first year of life [16], when structural and functional maturation of the kidney is still ongoing [9]. It is estimated that 2000 children undergo a nephrectomy annually in the United States [5,16]. The majority of these are due to non-oncological causes accounting for ~74% of pediatric nephrectomy, with the remainder due to oncological causes such as Wilm’s tumor [16]. Although limited, there are data to suggest that the kidney outcomes in EA-SFK may be worse than C-SFK [2,17]. For example, in the KIdney of MONofunctional Origin (KIMONO) study, individuals with an EA-SFK were associated with a greater incidence of renal injury compared with C-SFK [2].

We have an established ovine model of C-SFK, produced by fetal UNX in the sheep. As in the human, nephrogenesis is complete before birth in sheep. In sheep with a C-SFK, there is evidence of compensatory nephrogenesis with total nephron number 30% less (i.e., single SFK compared with two kidneys from sham animals) compared with sham sheep at ~130 days gestation [11]. By six months of age, sheep with a C-SFK have elevated arterial blood pressure [18,19], reduced GFR [20], reduced renal blood flow (RBF) [20], elevated urinary albumin excretion [19], and a kidney weight similar to the combined weight of both kidneys from sham-operated sheep [20]. We have also shown that sheep with a C-SFK have impaired nitric oxide (NO) bioavailability/production [21–24] and reduced renal functional reserve (RFR) [24–26] compared with sham counterparts. We hypothesize that the EA-SFK undergoes a greater degree of hyperfiltration compared with C-SFK owing to a greater nephron deficiency, contributing to greater and more progressive kidney injury.

The aim of the present study was to investigate whether, compared with C-SFK sheep, an EA-SFK, induced by UNX in lambs at four weeks of age, (1) increased blood pressure, glomerular hyperfiltration, and albuminuria, (2) reduced RFR in response to combined amino acid and dopamine (AA + D) infusion, (3) reduced kidney hemodynamic responses to NO synthase (NOS) inhibition via Nω-nitro-l-arginine methyl ester (L-NAME), and (4) reduced urinary total nitrate + nitrite (NOx) excretion as an index of NO production and bioavailability at eight months of age. The eight-month age time point in sheep corresponds to a comparable median age to develop renal injury (14.8 years old) in humans, observed in the KIMONO study in both EA-SFK and C-SFK, making it an appropriate age to investigate disease progression in these models [2].

## Methods

### Study approval

All experimental procedures were approved by an Animal Ethics Committee of Monash University (Ethics numbers: MARP/182/2016, #20476) and were performed in accordance with the guidelines of the National Health and Medical Research Council of Australia. The data in the sham and C-SFK groups, excluding the phase-contrast magnetic resonance imaging (PC-MRI) measurement of renal artery blood flow, have been reported in a previous study [24]. The EA-SFK experiments were conducted during the same period of time.

### Animals

Pure-bred merino sheep underwent three separate aseptic surgeries under general anesthesia: (1) to generate the experimental cohort pregnant merino ewes and their male fetuses underwent UNX (C-SFK; *n*=9), sham surgery (sham; *n*=7) at 100±3 days gestation (term; 150 days) or at 1±0.2 months of age male lambs underwent UNX to generate the early acquired group (EA-SFK; *n*=8); (2) at approximately five months of age, all lambs were surgically prepared with carotid arterial loops, allowing subsequent catheterization for measurement of blood pressure (BP; mean, systolic, diastolic), heart rate (HR), and blood sampling, as previously detailed [19]; (3) at eight months of age, a bladder catheter was surgically inserted into the bladder to facilitate urine collection for assessment of kidney function experiments, as previously described [27].

The fetal UNX surgery has been previously described in detail [23,24]. To generate the EA-SFK group at ∼1±0.2 months of age, male lambs underwent UNX surgery. Analgesia was provided by a transdermal fentanyl patch applied to the inguinal groin region the day before surgery (Durogesic 12.5 µg/h; release ∼0.002 mg/kg/h; Jansen, removed 72 h after surgery). On the day of surgery, lambs were anesthetized with an intravenous (i.v.) infusion of sodium thiopentone (10–13 mg/kg; Jurox) and after endotracheal intubation, maintained with isoflurane (0–5% in 100% O_2_). Following aseptic preparation and prior to incision, bupivacaine (2 mg/kg, 0.5% with adrenaline, AstraZeneca) was applied to the incision site. The lamb was positioned in lateral recumbency; an incision was made over the left flank, the left kidney was located, and the surrounding fat was cleared off. Following the ligation of the left renal artery, vein, and ureter, the kidney was excised. During the anesthetic period, i.v. fluids (Hartmann solution; 10 ml/kg per hour; Freeflex) were administered, and temperature and HR were monitored regularly. Lambs were treated with intramuscular antibiotic (20 mg/kg ampicillin sodium; Mylan) at surgery and once daily for three days postsurgery and allowed to recover from surgery. All lambs remained with their mothers until weaning at 16 weeks of age. Animals were housed at the Monash University’s Gippsland Field Station in between surgical/experimental procedures or at Monash Animal Research Platform (MARP) during surgical/experimental procedures. Sheep remained at MARP from approximately five months of age.

### MRI

#### Kidney volume

Sheep underwent MRI, as previously detailed [24]. At two and six months of age, kidney volume was measured using a three-dimensional T1 VIBE DIXON sequence using a 3T Siemans Skyra (Siemens, Erlangen, Germany) under general anesthesia.

### Phase-contrast renal artery blood flow

At two months of age, following the measurement of kidney volume, sheep underwent PC-MRI to assess RBF. Identification of the renal artery was performed using localizer images of the renal artery in three planes. The axial acquisition was carefully positioned perpendicular to the sagittal and coronal long axes of the renal artery at a location proximal to bifurcations of the renal artery. A cine PC-MRI was performed using a retrospective ECG synchronization. The PC-MRI parameters for renal arterial blood flow were as follows: repetition time, 20.36 ms; echo time, 2.92 ms; flip angle, 20°; phases, 50; and a flow-encoded maximum velocity of ∼110 cm/sec depending on the velocity of flow through the renal artery. Renal artery blood flow analysis was performed by Monash Biomedical Imaging radiographers using the ARGUS flow software (Siemens, Erlangen, Germany). Renal artery blood flow (ml/min) was corrected for body weight (kg) and kidney volume (cm^3^). As both C-SFK and EA-SFK sheep had only the right kidney, RBF was performed in the right kidney of the sham animals and presented here as a single kidney blood flow.

### Experimental protocol

At eight months of age, a Foley catheter (size 10–12F; Bard Australia) was surgically inserted under general anesthesia, to allow urine sampling and collection to measure kidney function, as previously described [27]. Sheep were individually housed in metabolic cages and allowed to recover from surgery for three to four days. Before the experimental period and under local anesthetic (subcutaneous, 20 mg/kg, Lignocaine with 0.5% adrenaline), an indwelling carotid arterial catheter was inserted into the carotid loop and connected to a pressure transducer (Argon pressure transducer, LivaNova Australia Pty Ltd) that allowed continuous measurement of conscious BP (systolic, diastolic, mean), as previously described [19]. In addition, to facilitate infusion, a polyethylene catheter was inserted into the jugular vein (PE tubing, 1.2 × 1.0, Microtube Extrusions, Australia).

### Basal cardiovascular and kidney function

Basal cardiovascular and kidney function were measured over a 3-h period by clearance of ^51^Chromium ethylenediaminetetraacetic acid (^51^Cr EDTA) and para-aminohippuric acid (PAH) administered (i.v.) to assess GFR and RBF, respectively [28]. Following 1 h of equilibration, urine was collected at 20-min intervals, and blood samples (3 ml) were collected at the mid-point of each urine collection. Both BP and HR were measured continuously during this 3-h period.

### Cardiovascular and kidney function in response to AA+D infusion

On a separate day, after a 1-h equilibration period, basal BP, HR, and kidney function were measured for 60 min. RFR was measured by the i.v. infusion of amino acids (0.065 ml/kg per hour of a 10% solution without electrolytes, Synthamin 17; Baxter) and dopamine (5 μg/kg per minute, i.v., dopamine hydrochloride; Sigma-Aldrich) for 2 h.

### Cardiovascular and kidney function in response to L-NAME

On a separate day, after a 1-h equilibration period, basal BP, HR, and kidney function were measured for 60 min. Subsequently, BP, HR, and kidney function were determined during 2 h of i.v. infusion of L-NAME (20 mg/kg bolus plus 20 mg/kg per hour infusion for 2 h, L-NAME hydrochloride; Cayman Chemical). Sheep were euthanized with an overdose of pentobarbitone sodium (100 mg/kg, i.v.) within two to three days of experimentation,

### Sample analysis

^51^Cr EDTA levels were determined with a gamma counter (PerkinElmer Wizard 1470) and PAH concentration assessed with a rapid microplate assay method [29]. RBF, renal vascular resistance (RVR), and filtration fraction were calculated using standard formulae [30]. Basal albumin excretion was measured via an Albuwell O-ovine microalbuminuria ELISA kit (Exocell, Philadelphia, PA) in a 20-min urine sample (expressed as mg/24 h), as per the manufacturer’s instructions. Basal total urinary NOx concentration was measured via colorimetric assay (780001: Cayman Chemical Company) as per the manufacturer’s instructions. Plasma renin activity (PRA) was determined by radioimmunoassay (ProSearch International Pty, Malvern, Australia).

### Kidney histopathology

Kidney samples were flushed with saline and fixed in 4% paraformaldehyde, with one quarter of a midline kidney slice processed to paraffin, sectioned at 4 μm, and stained with Masson trichrome and Periodic acid–Schiff (PAS) stain by Monash Histology Platform. Using the Aperio ImageScope software, the Positive Pixel Count Algorithm optimized for Masson trichrome was used to distinguish collagen in the renal cortex. To measure glomerular area, at least 30 glomeruli were traced in PAS-stained kidneys when the vascular pole was evident. Histopathology scoring for tubular injury, defined as atrophy, degeneration or thickening of tubular basement membrane, PAS+casts, and tubular dilation were performed in PAS-stained kidneys. All analyses were performed blinded to treatment groups.

### Statistical analysis

All values are presented as mean±SEM. Statistical analysis was performed using GraphPad Prism 10 (GraphPad Inc., CA, U.S.A.), and the level of statistical significance was accepted as *P*≤0.05. Data were tested for normality via a Shapiro–Wilk test; if data violated normality, a rank-based test was used. A mixed model ANOVA examining the effect of factors—group (*P*_group_; sham, C-SFK, or EA-SFK), age (*P*_age_), and their interaction (*P*_group×age_)—was used to assess body weight and kidney volume. A one-way ANOVA was used for all other variables, and where appropriate, a Tukey’s post hoc analysis was performed comparing each group. A Bartlett’s test was used to assess the equality of variance between groups, and if the variance was not equal, a Welch ANOVA was used with a Dunnett’s T3 post hoc analysis performed as appropriate.

## Results

### Body weight

**Figure 1:**
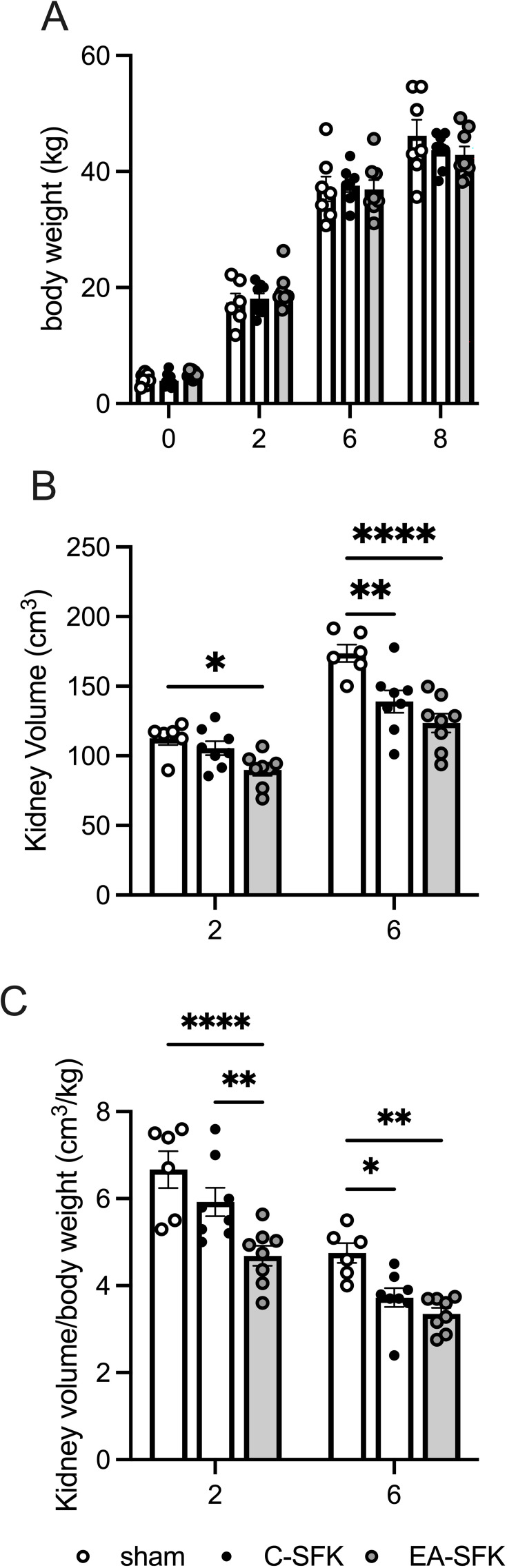
Body weight and kidney volume throughout study. Data presented in male lambs that underwent fetal sham surgery (sham; presented as two kidneys for kidney volume data), fetal uninephrectomy (C-SFK), and uninephrectomy at four weeks of age (EA-SFK). Data are mean±SEM. (**A**) Body weight at birth (0 months) and at two, six, and eight months of age. Sham, *n*=6/7 per age; C-SFK, *n*=8/9 per age; EA-SFK, *n*=8 (**B**) Kidney volume (cm^3^) and (**C**) kidney volume normalized to body weight (cm^3^/kg) at two and six months of age. For two to six months, sham, *n*=6; C-SFK, *n*=8; EA-SFK, *n*=8. *P* values are from Tukey’s post hoc test after a two-way mixed effect ANOVA. *P<0.05 , **P<0.01 , ****P<0.0001. C-SFK, congenital solitary functioning kidney; EA-SFK, early acquired solitary functioning kidney.

Birth weights were similar between groups, and body weights were similar between groups at two, six, and eight months of age (Figure 1a).

### Kidney volume (MRI)

Estimated total kidney volume in sham animals is presented as two kidneys and in C-SFK and EA-SFK animals as one kidney (Figure 1b–c). At two months of age, total kidney volume was ∼20% lower in EA-SFK sheep compared with sham sheep (*P*=0.038, Figure 1b) but was similar between sham and C-SFK sheep and similar between C-SFK and EA-SFK sheep (Figure 1b). At six months of age, total kidney volume was ∼20% lower in C-SFK sheep compared with sham (*P* = 0.001), ∼29% lower in EA-SFK sheep compared with sham (*P* < 0.0001), and similar between C-SFK and EA-SFK groups (Figure 1b).

At two months of age, total kidney volume normalized to body weight was ∼29% lower in EA-SFK sheep compared with sham (*P*<0.0001), ∼21% lower in EA-SFK sheep compared with C-SFK (*P*=0.004; Figure 1c), and similar between C-SFK and sham groups (Figure 1c). At six months of age, total kidney volume normalized to body weight was ∼22% lower in C-SFK sheep compared with sham (*P*=0.03), ∼29% lower in EA-SFK sheep compared with sham (*P*=0.002), and similar between C-SFK and EA-SFK groups (Figure 1c).

### Renal artery blood flow (PC-MRI)

**Figure 2:**
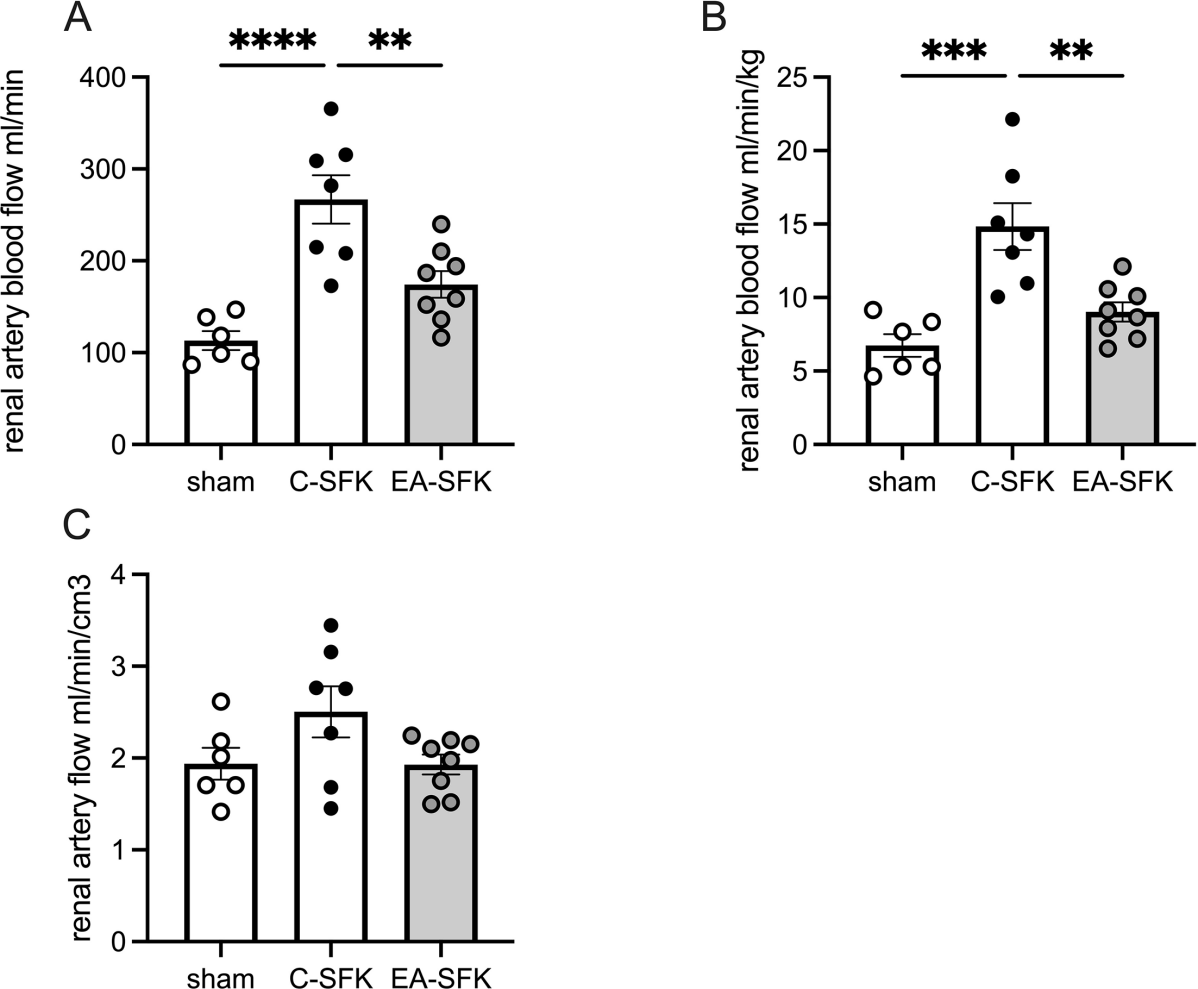
Magnetic resonance imaging (MRI) phase-contrast renal artery flow at two months of age. (**A**) Renal artery blood flow (ml/min), (**B**) renal artery blood flow normalized to body weight (ml/min/kg), and (**C**) renal artery blood flow normalized to kidney volume (ml/min/cm³) at two months of age. Data presented in male lambs that underwent fetal sham surgery (sham; *n*=6 presented as a single kidney renal blood flow), fetal uninephrectomy (C-SFK, *n*=7), or uninephrectomy at four weeks of age (EA-SFK; *n*=8). Data are mean±SEM. *P* values are from Tukey’s post hoc test after one-way ANOVA. **P<0.01, ***P<0.001, ****P<0.0001. C-SFK, congenital solitary functioning kidney; EA-SFK, early acquired solitary functioning kidney.

Renal artery blood flow in sham animals is presented as flow per one kidney. At two months of age, renal artery blood flow (ml/min) was ∼135% greater in C-SFK sheep compared with a single kidney of sham animals (*P*<0.0001, Figure 2a), ∼35% lower in EA-SFK sheep compared with C-SFK (*P*=0.005, Figure 2a), and similar between sham and EA-SFK groups. Renal artery blood flow corrected for body weight was greater in C-SFK sheep compared with sham counterparts (∼8.1 ml/min/kg, *P*=0.0002, Figure 2b), lower in EA-SFK sheep compared with C-SFK (∼5.8 ml/min/kg, *P*=0.003, Figure 2b), and similar between sham and EA-SFK groups. When renal artery blood flow was corrected by kidney volume (cm^3^), it was similar between groups (Figure 2c).

### Basal mean arterial pressure, HR, albumin excretion, and PRA at eight months of age

Mean arterial pressure (MAP) was ~12 mm Hg higher in C-SFK animals compared with sham (*P*=0.0005), ~ 9 mm Hg lower in EA-SFK animals compared with C-SFK (*P*=0.004), and similar between sham and EA-SFK groups (Table 1). HR was similar between all groups (Table 1). Urinary albumin excretion was ~67% greater in C-SFK compared with sham sheep (*P*=0.02), significantly lower in EA-SFK sheep compared with C-SFK (~34%; *P*=0.048), and similar between sham and EA-SFK animals (Table 1). Compared with sham sheep, PRA was significantly lower in C-SFK sheep (*P*=0.02) but was similar between sham and EA-SFK sheep and between C-SFK and EA-SFK sheep (Table 1).

**Table 1:** Basal cardiovascular and kidney parameters at eight months of age

Variable	Sham (*n*=7)	C-SFK (*n*=9)	EA-SFK (*n*=8)
MAP (mm Hg)	81±1	93±1^2^	84±3^4^
Heart rate (beats/min)	80±2	83±2	79±2
Albumin excretion (mg/24 h)	92.5±7.1	154.1±15.9^1^	102.4±17.2^3^
PRA (ng/ml/h)	2.2±0.1	1.3±0.1^1^	1.9±0.3

Data are presented as mean±SEM.

1 *P*<0.05 comparing between sham and C-SFK groups analyzed via a one-way ANOVA followed by a Tukey’s post hoc

2 *P*<0.001 comparing between sham and C-SFK groups analyzed via a one-way ANOVA followed by a Tukey’s post hoc

3 *P*<0.05 comparing between C-SFK and EA-SFK groups analyzed via a one-way ANOVA followed by a Tukey’s post hoc

4 *P*<0.001 comparing between C-SFK and EA-SFK groups analyzed via a one-way ANOVA followed by a Tukey’s post hoc

MAP, mean arterial pressure. PRA, plasma renin activity.

### Basal kidney function at eight months of age

**Figure 3:**
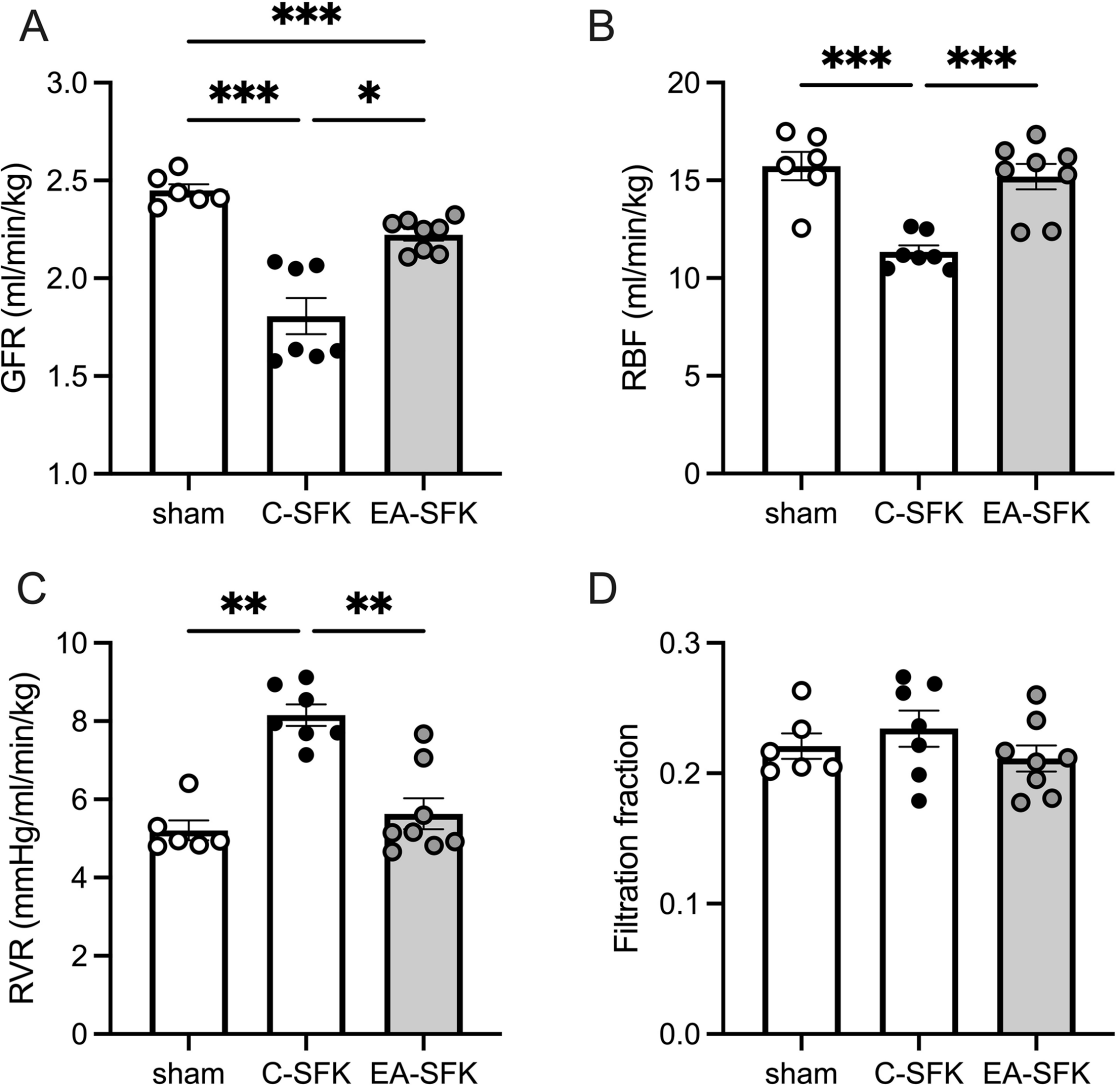
Basal kidney hemodynamics at eight months of age. (**A**) Basal glomerular filtration rate (GFR), (**B**) renal blood flow (RBF), (**C**) renal vascular resistance (RVR), and (**D**) filtration fraction measured over 3 h presented as a singular average of this period. Data presented in male lambs that underwent fetal sham surgery (sham, *n*=6 , fetal uninephrectomy (C-SFK, *n*=7), or uninephrectomy at four weeks of age (EA-SFK, *n*=8). Data are mean±SEM. *P* values are from Tukey’s post hoc test after one-way ANOVA for parametric data (**B, D**), from Kruskal–Wallis post hoc for nonparametric data, (**C**) and a Dunnett’s T3 post hoc following a Welch ANOVA for uneven SD (**A**). *P<0.05 **P<0.01 , ***P<0.001. C-SFK, congenital solitary functioning kidney; EA-SFK, early acquired solitary functioning kidney.

GFR compared with sham sheep was lower in C-SFK (~26%, *P*=0.0009) and EA-SFK sheep (~9%, *P*=0.0008; Figure 3a). GFR was greater in EA-SFK than C-SFK sheep (~23%, *P*=0.01; Figure 3a). RBF was lower (~28%, *P*=0.0003) in C-SFK animals compared with sham, greater in EA-SFK animals compared with C-SFK (~34%, *P*=0.0005), and similar between sham and EA-SFK groups (Figure 3b). RVR was greater in C-SFK sheep (~57%, *P*<0.0001) compared with sham counterparts, lower in EA-SFK sheep (~31%, *P*=0.0006) compared with C-SFK counterparts, and similar between sham and EA-SFK groups (Figure 3c). Filtration fraction was similar between groups (Figure 3d).

### Cardiovascular and kidney function in response to combined AA+D infusion

**Figure 4:**
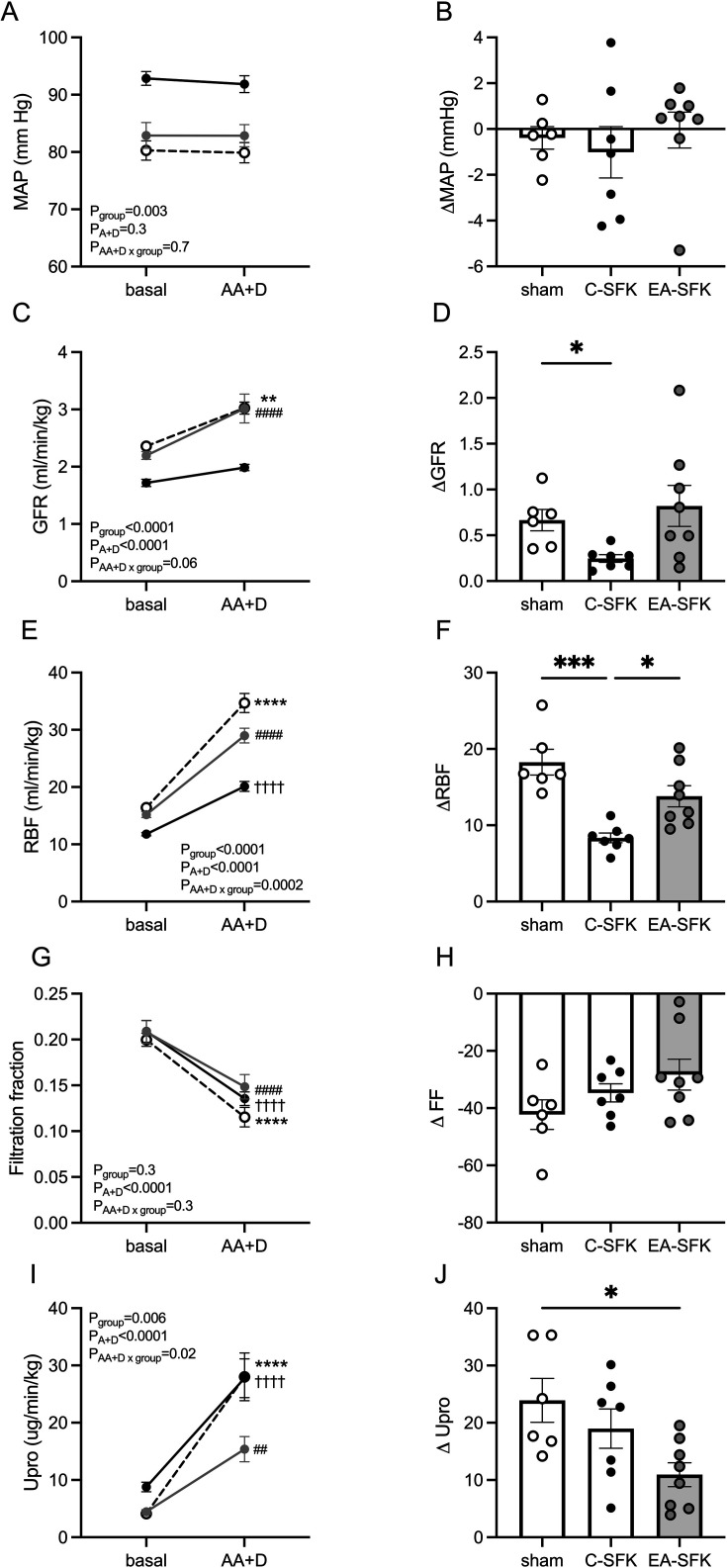
Cardiovascular and kidney hemodynamics responses to amino acid and dopamine (AA+D) infusion at eight months of age. (**A, B**) MAP, (**C, D**) GFR, (**E, F**) RBF, (**G, H**) FF, and (**I, J**) urinary protein excretion in response to AA+D infusion. Data presented in male lambs that underwent fetal sham surgery (sham, *n*=6), fetal uninephrectomy (C-SFK, *n*=7), or uninephrectomy at four weeks of age (EA-SFK, *n*=8) . Data are mean±SEM. Absolute data analyzed via a two-way repeated measures ANOVA followed by a Tukey’s post hoc test as appropriate (**A, C, E, G, I**). **P<0.01 ****P<0.0001 comparing baseline with AA+D in sham, ††††*P*<0.0001 comparing baseline with AA+D in C-SFK, ##*P*<0.01, ####*P*<0.0001 comparing baseline with AA+D in EA-SFK. Change from baseline (**A**) data analyzed via a one-way ANOVA followed by a Tukey’s post hoc test (**B, F, H, J**) and a Dunnett’s T3 post hoc following a Welch ANOVA for uneven SD (**D**). *P<0.05 , ***P<0.001. C-SFK, congenital solitary functioning kidney; EA-SFK, early acquired solitary functioning kidney; GFR, glomerular filtration rate; MAP, mean arterial pressure; RBF, renal blood flow.

AA+D infusion did not significantly affect MAP (Figure 4a and b) or HR in any group. AA+D caused an increase in RBF and GFR, which led to a decrease in filtration fraction in all groups (P_AA+D_<0.0001, Figure 4c, g and e). In response to AA+D infusion, GFR was not significantly different from baseline in C-SFK sheep (Figure 4c) but increased in sham (∼0.67 ml/min/kg, *P*=0.0013, Figure 4d) and EA-SFK sheep (∼0.82 ml/min/kg, *P*<0.0001, Figure 4d) from baseline. AA+D caused a blunted increase in RBF in C-SFK sheep compared with sham (∼9.9 ml/min/kg less, *P*=0.0002, Figure 4f), greater increase in EA-SFK sheep compared with C-SFK sheep (∼5.5 ml/min/kg greater, *P*=0.02, Figure 4f), and a trend towards a lesser increase in RBF in EA-SFK sheep compared with sham (∼4.47 ml/min/kg less, *P*=0.06, Figure 4f). The fall in filtration fraction and increase in urine flow were not significantly different between groups in response to AA+D infusion. AA+D caused an increase in urinary protein excretion, which was blunted in EA-SFK sheep (∼12.97 ug/min/kg less, *P*=0.02, Figure 4j) compared with sham and similar between sham and C-SFK groups and C-SFK and EA-SFK groups (Figure 4j) in response to AA+D infusion.

### Cardiovascular and kidney function in response to L-NAME

**Figure 5:**
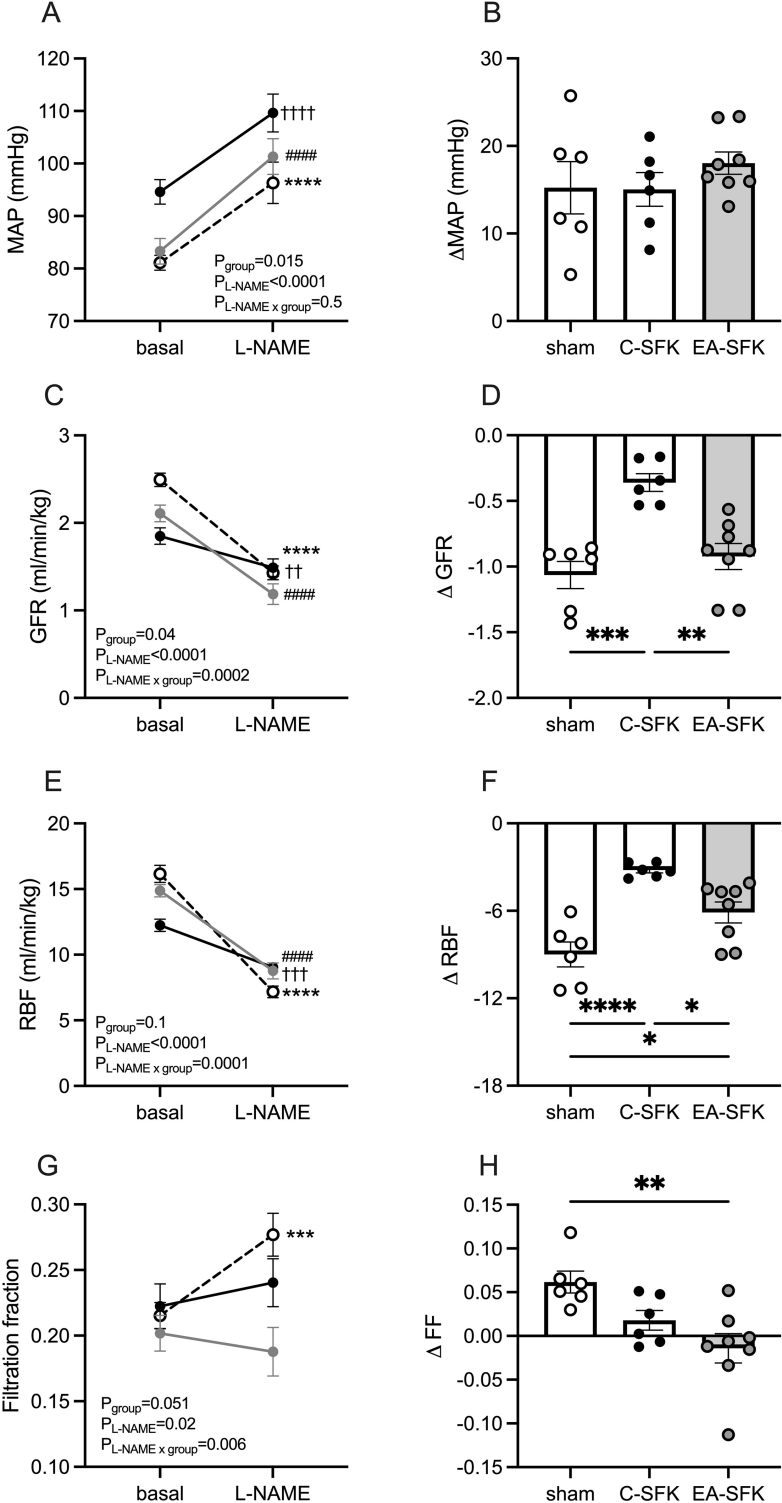
Cardiovascular and kidney hemodynamic responses to NOS inhition at eigtht months of age. (**A, B**) MAP, (**C, D**) GFR, (**E, F**) RBF, and (**G, H**) FF in response to L-NAME. Data presented in male lambs that underwent fetal sham surgery (sham, *n*=6), fetal uninephrectomy (C-SFK, *n*=6), or uninephrectomy at four weeks of age (EA-SFK, *n*=8). Data are mean±SEM. Absolute data analyzed via a two-way repeated measures ANOVA followed by a Tukey’s post hoc test as appropriate (**A, C, E, G**). ***P<0.001, ****P<0.0001 comparing baseline with L-NAME in sham, ††*P*<0.01,†††*P*<0.001, ††††*P*<0.0001 comparing baseline with L-NAME in C-SFK, #### *P*<0.0001 comparing baseline with L-NAME in EA-SFK. Change from baseline (**A**) data analyzed via a one-way ANOVA followed by a Tukey’s post hoc test (**B, F, H, J**), *P<0.05 , **P<0.01, ***P<0.001. C-SFK, congenital solitary functioning kidney; EA-SFK, early acquired solitary functioning kidney; GFR, glomerular filtration rate; MAP, mean arterial pressure; RBF, renal blood flow.

In response to L-NAME, the increase in MAP and decrease in HR from baseline were not different between groups (Figure 5a and b). L-NAME caused GFR and RBF to decrease from baseline in all groups (P_L-NAME_<0.0001, Figure 5c and e). The fall in GFR was blunted in C-SFK sheep compared with sham (∼0.70 ml/min/kg less, *P*=0.0003, Figure 5d), greater in EA-SFK sheep compared with C-SFK (∼0.56 ml/min/kg greater, *P*=0.0014, Figure 5d), and similar between EA-SFK and sham sheep in response to L-NAME (Figure 5d). L-NAME caused a reduced fall in RBF in C-SFK sheep compared with sham (∼5.8 ml/min/kg less, *P*<0.0001, Figure 5f), reduced fall in RBF in EA-SFK sheep compared with sham (∼2.8 ml/min/kg less, *P*=0.02, Figure 5f), and greater fall in RBF in EA-SFK compared with C-SFK (∼2.9 ml/min/kg less, *P*=0.02, Figure 5f). L-NAME caused a greater fall in RBF than GFR, resulting in an increase in FF from baseline in sham sheep (*P*=0.0009, Figure 5g). However, in both EA-SFK and C-SFK sheep, L-NAME caused a similar fall in RBF and GFR resulting in FF that was similar to baseline (Figure 5g).

### Basal total urinary NOx excretion

**Figure 6:**
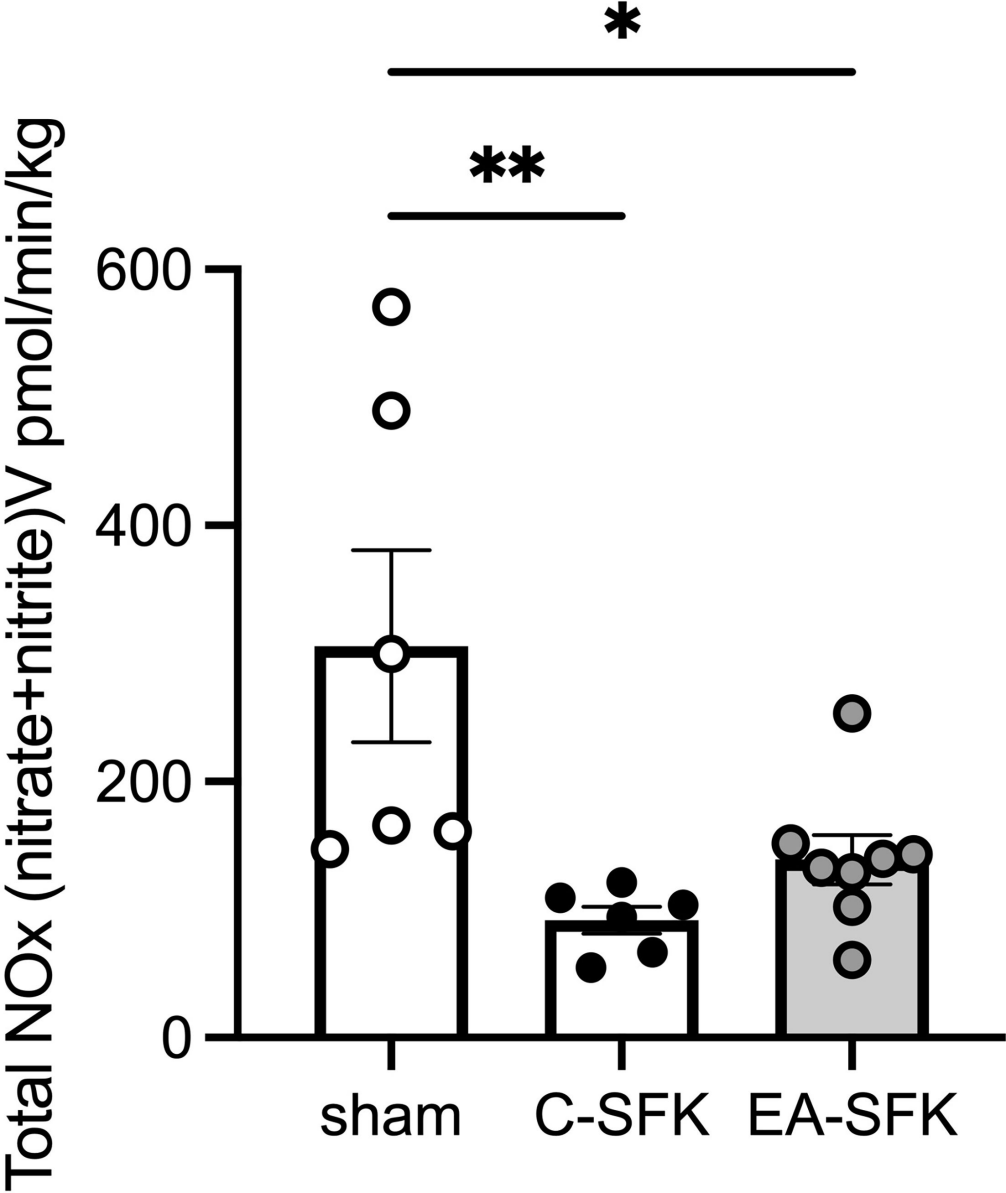
Basal total urinary NOx at eight months of age. Data presented in male lambs that underwent fetal sham surgery (sham, *n*=6) , fetal uninephrectomy (C-SFK, *n*=6) , or uninephrectomy at four weeks of age (EA-SFK, *n*=8) . Data are mean±SEM. *P* values are from Tukey’s post hoc test after one-way ANOVA. *P<0.05 **P<0.01. C-SFK, congenital solitary functioning kidney; EA-SFK, early acquired solitary functioning kidney.

Basal total urinary NOx excretion was ∼70% lower in C-SFK sheep and ∼54% lower in EA-SFK sheep compared with sham counterparts (*P*=0.008 and *P*=0.03, respectively; Figure 6). Basal urinary NOx excretion was similar between EA-SFK and C-SFK groups.

### Body and organ weights at eight months of age

Body weights were similar between groups(Table 2). Total kidney volume and kidney volume corrected for body weight were also similar between groups(Table 2). Heart weight and heart weight normalized to body weight were significantly greater in C-SFK animals compared with sham animals(Table 2). Heart weight was similar between sham and EA-SFK groups and between EA-SFK and C-SFK groups(Table 2). Heart weight normalized to body weight was significantly greater in EA-SFK sheep compared with sham and similar between EA-SFK and C-SFK sheep )(Table 2).

**Table 2:** Body and organ weights at eight months of age

Variable	Sham (*n*=7)	C-SFK (*n*=9)	EA-SFK (*n*=8)
Body weight (kg)	46±3	44±1	43±1
Total kidney weight (g)	123±4	115±9	97±7
Normalized kidney weight (g/kg)	2.7±0.2	2.6±0.2	2.3±0.1
Heart weight (g)	122±7	153±11^1^	144±6
Normalized heart weight (g/kg)	2.6±0.2	3.5±0.2^2^	3.4±0.1^3^

Data are presented as mean±SEM. Note that data for heart weight (g) and normalized heart weight (g/kg) are representative of sham, *n*=6; C-SFK, *n*=7; EA-SFK, *n*=8.

1 *P*<0.05 comparing between sham and C-SFK groups analyzed via a one-way ANOVA followed by a Tukey’s post hoc or a Kruskal–Wallis test followed by a Dunn’s post hoc for nonparametric data

2 *P*<0.01 comparing between sham and C-SFK groups analyzed via a one-way ANOVA followed by a Tukey’s post hoc or a Kruskal–Wallis test followed by a Dunn’s post hoc for nonparametric data

3 *P*<0.05 comparing between sham and EA-SFK groups analyzed via a one-way ANOVA followed by a Tukey’s post hoc or a Kruskal–Wallis test followed by a Dunn’s post hoc for nonparametric data

### Kidney histopathology

**Figure 7:**
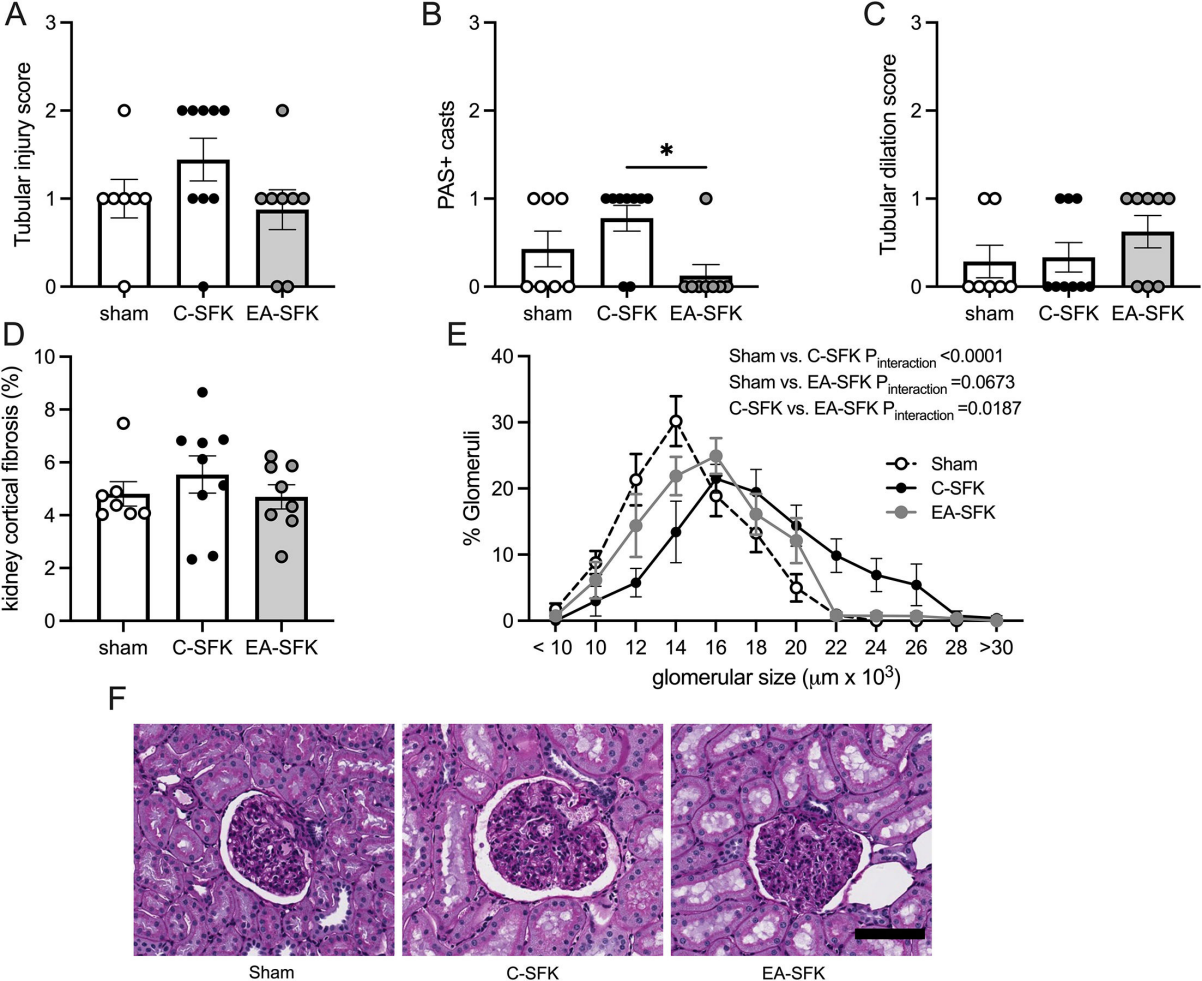
Kidney histopathology and morphometry at eight months of age. **(A**) Tubular injury score, (**B**) PAS+casts, (**C**) tubular dilation score, (**D**) kidney cortical fibrosis (% collagen), (**E**) glomerular size distribution (% glomeruli vs. glomerular size (µm²x10^3^). (**F**) Representative micrograph images of glomeruli; scale bar is 100 µm. Data presented in male lambs that underwent fetal sham surgery (sham; *n*=7), fetal uninephrectomy (C-SFK, *n*=9 , or uninephrectomy at four weeks of age (EA-SFK; *n*=8). Data are mean±SEM. *P* values are from Tukey’s post hoc test after one-way ANOVA (**A, B, C, D**) or from a two-way ANOVA (**E**). *P<0.01. C-SFK, congenital solitary functioning kidney; EA-SFK, early acquired solitary functioning kidney.

Tubular injury score, tubular dilation score, and the percent of kidney cortical fibrosis were similar between groups (Figure 7a, c and d). PAS positive casts were not different between sham and C-SFK sheep or between EA-SFK and sham sheep but were significantly less in EA-SFK sheep compared with C-SFK sheep (*P*=0.017, Figure 7b). Glomerular area distribution was shifted rightward in C-SFK sheep compared with sham (*P*_interaction_<0.0001, Figure 7e) and compared with EA-SFK sheep (*P*_interaction_=0.0187, Figure 7e), indicating C-SFK sheep had larger glomeruli. There was only a trend toward a rightward shift in glomerular area distribution in EA-SFK sheep compared with sham sheep (*P*_interaction_=0.0673, Figure 7e).

## Discussion

The main finding of this study was that despite evidence of hyperfiltration (increased single nephron GFR), in a sheep model of EA-SFK produced by UNX at four weeks of age, high BP, albuminuria, and reduced kidney function were absent at eight months of age. These findings do not support our hypothesis that EA-SFK sheep would have a greater loss of kidney function associated with greater hyperfiltration than C-SFK sheep. However, the rate of renal hypertrophy and degree of hyperfiltration were greater in the EA-SFK than in the C-SFK model, which may be a prelude to a decline in renal function with increasing age.

Loss of a kidney early in life caused marked renal hypertrophy in sheep models of EA-SFK and C-SFK. Kidney masses in both EA-SFK and C-SFK groups were comparable to sheep with two kidneys at eight months of age. However, at two months of age, the EA-SFK sheep had reduced kidney volume corrected for body weight, measured *in vivo* via MRI, compared with C-SFK sheep. At the same time, RBF corrected for body weight, measured *in vivo* via MRI, was also less in EA-SFK sheep compared with C-SFK. In comparison, C-SFK sheep had greater RBF at two months of age and renal mass was similar to sham animals with two kidneys. An increase in RBF following UNX has been suggested to be an initiating factor in the hypertrophic growth of the remaining kidney [8]. The elevation in RBF in response to renal mass reduction may be mediated by NO [31], with sheer stress increasing NO production [32]. Indeed, the increase in RBF and hypertrophy of the remaining kidney were blunted by NO blockade with L-NAME following UNX in the adult rat [33]. Similarly, in eNOS knockout mice, the increase in RBF and kidney hypertrophy were absent following UNX [31]. However, the reduced level of kidney hypertrophy and a lesser increase in RBF at two months of age in sheep with an EA-SFK likely reflect the time since nephrectomy, being 3.5 months in the C-SFK, and one month in the EA-SFK model.

In the present study, by eight months of age, kidney weight was similar between C-SFK and EA-SFK sheep. Our previous studies indicate that at this age renal growth has plateaued in the C-SFK [25,34]. It remains to be seen if renal growth has reached a maximum in the EA-SFK sheep. Certainly, it appears that kidney growth has been more rapid in the EA-SFK group. Also, at eight months of age, RBF, measured via clearance of PAH, was higher in EA-SFK sheep compared with C-SFK and lower in C-SFK compared with sham, as previously shown [24]. Additionally, by eight months of age, NO bioavailability was reduced in both EA-SFK and C-SFK sheep compared with sham sheep. This was indicated by reduced NOx urinary excretion and RBF response to NOS inhibition in both SFK groups compared with the sham group. However, at this age, the deficit in NO bioavailability was greater in C-SFK than the EA-SFK sheep. The deficit in NO bioavailability observed in EA-SFK sheep and C-SFK is likely not associated with changes in kidney cortical eNOS and phosphorylated eNOS protein expression, which have previously been shown to be similar between C-SFK and sham sheep [24]. Elevated asymmetric dimethylarginine (ADMA), a competitive NOS inhibitor and independent inducer of reactive oxygen species, is observed in pediatric [35,36] and adult CKD [37]. It is possible that elevated ADMA and/or reactive oxygen species could be interfering with NO bioavailability in both SFK models. Taken together, this may indicate that alterations in NO early in life in C-SFK and/or EA-SFK may initially increase kidney hypertrophy and RBF, but a deficit in NO bioavailability may precede kidney functional decline. However, further studies exploring the contribution of NO to kidney function in the early postnatal period in SFK and as kidney function declines would be required to further understand the role of NO in hyperfiltration mediated injury.

GFR is the sum of filtration of each individual nephron. Glomerular hyperfiltration is an adaptative response to the loss of nephrons leading to increased single nephron GFR; although GFR can be maintained within normal ranges initially, over time is associated with impairment of glomerular filtration barrier integrity, initiation of albuminuria, and kidney functional decline [8]. It is also evident that the timing and duration of hyperfiltration affect the degree of kidney injury [38]. In rats, it has been shown that UNX at 5, 12, and 40 days of life results in a ∼115%, ∼74%, and ∼37% increase in single nephron GFR, respectively, suggesting that the degree of hyperfiltration is contingent on the timing of kidney loss [39]. In the present study, basal GFR was ∼23% greater in the EA-SFK group compared with the C-SFK group and ∼9% less in the EA-SFK group compared with sham group. This indicates that EA-SFK sheep were hyperfiltering at the level of the single nephron maintaining a GFR close to sham levels, despite 50% less nephrons and is supported by a similar RBF between EA-SFK and sham groups. This hyperfiltration may be mediated by an increase in filtration surface area and/or an increase in glomerular pressure. However, given that glomerular area was less in EA-SFK sheep compared with C-SFK, this would suggest that an increase in glomerular pressure is mediating hyperfiltration and leads to degradation of the filtration barrier [8]. The EA-SFK sheep also had similar albumin excretion as sham animals, suggesting that the hyperfiltration in these animals had not induced glomerular injury. Comparatively, as previously shown [24], C-SFK sheep demonstrate elevated albuminuria and reduced kidney function (GFR, RBF, elevated RVR) compared with sham sheep. This suggests that in this sheep model, the C-SFK group is likely further advanced in the course of hyperfiltration-mediated injury compared with EA-SFK sheep. In Wilm’s tumor survivors that underwent UNX and long-term (∼25 years) follow-up, GFR decreases with longer-term follow-up time in these individuals [40]. At eight months of age, it is notable that the renal growth trajectory was accelerated and the degree of hyperfiltration was enhanced in the EA-SFK model compared with the C-SFK group. Whether this level of hyperfiltration would induce kidney injury in EA-SFK sheep in the future requires longer-term follow-up.

In this study, the EA-SFK group had a similar RFR as sham animals. This suggests that despite hyperfiltration, RFR was not impaired in the EA-SFK animals. The absence or impairment of RFR has been suggested to be an early marker of hyperfiltration, with a greater degree of hyperfiltration proposed to result in a greater reduction in RFR [41–43]. In a small study in children with SFK and normal basal renal function, it has been shown that RFR was reduced in ~50% of children in response to an oral protein load [43]. This suggests that RFR may be able to detect hyperfiltration in some, but not all, children with SFK. In patients with CKD RFR, induced by i.v., infusion of 10% mixed amino acids was normal at CKD stage 1 and reduced from CKD stage 2 onward [44]. This may indicate that more established CKD is required to detect impairment in RFR and support our finding that in the C-SFK sheep [24,25], which have reduced GFR and albuminuria, RFR is blunted compared with sham sheep at 8 and 20 months of age. Further studies are needed to establish if RFR reduces with age in EA-SFK sheep. In response to an infusion of amino acids or protein load, the rise in plasma proteins results in a rise in urinary excretion of protein [45,46]. In the present study, urinary protein excretion in response to amino acid and dopamine infusion was blunted in EA-SFK sheep compared with sham sheep. Reabsorption of filtered proteins occurs in the proximal tubule via megalin-cubulin complex [47]. In rats, proximal tubule length increases (~35−70%) following UNX along with increased expression of megalin and cubulin, which may increase the reabsorption of proteins by the proximal tubule and eventually lead to proximal tubular injury [47–49]. Studies have explored the utility of a tubular stress test in kidney donors and have shown that tubular secretion of creatine in response to a protein meal or infusion of creatinine is reduced in kidney donors [50–52]. Whether assessment of tubular function might be a possible alternative to assess risk of kidney injury in children with SFK requires further investigation.

In the present study, EA-SFK sheep were normotensive, measured via an indwelling carotid catheter in unanesthetized animals. Despite this, EA-SFK sheep had increased heart weight corrected for body weight compared with sham sheep. In adult normotensive humans, an increase in left ventricular mass is associated with the development of hypertension [53]. Similarly, in spontaneously hypertensive rats, left ventricular hypertrophy precedes an elevation in BP [54]. Therefore, this may suggest that in EA-SFK sheep, the increased heart weight may be a precursor to high BP. A higher incidence of kidney injury has been observed in children with EA-SFK compared with C-SFK [2]. Moreover, EA-SFK children have been observed to have ∼11% lower GFR than C-SFK children [17]. However, in both these studies, EA-SFK children were older than C-SFK [2,17], and older age was found to be a risk factor for kidney injury [2]. Therefore, studies on the long-term impact of a C-SFK or EA-SFK are still needed, especially as they age.

Strengths of this study include the similarity of kidney development in sheep and humans, and that our measurements were performed in conscious animals, except MRI measurements. A limitation of the current study is that we could not serially measure BP and kidney function from early in life because bladder catheterization is challenging in male sheep. Additionally, given the different periods since nephrectomy (7 months EA-SFK; 9.5 months C-SFK), longer-term follow-up of the EA-SFK sheep would strengthen the conclusions that could be drawn about the trajectory of disease in these animals. Another limitation is that the present study was only performed in male sheep. While unilateral renal agenesis is more commonly identified in males (63%) than females [15], there is no sex difference in the incidence of paediatric UNX [16]. Evidence in animal models suggests that female sex may confer cardio and renal protection in C-SFK. In our previous studies in female sheep with a C-SFK and intact ovaries, elevations in BP are not observed until 24 months of age [55]. Comparatively, both male C-SFK sheep and ovariectomized female C-SFK sheep develop elevated BP earlier (six months of age) [18,19], suggesting that there may be a cardio-protective role of sex hormones in female sheep. Future studies examining sex differences in both SFK models and whether treatments are more or less effective in females with SFK are warranted.

In conclusion, in a sheep model of EA-SFK, despite marked hyperfiltration resulting in normalization of GFR and RFR, both albuminuria and high BP were not present at eight months of age. However, there were signs of cardiac hypertrophy and vasodilator imbalance that may herald a decline in kidney function.

**Clinical Perspectives**

- Being born with one kidney or acquiring a solitary functioning kidney (SFK), via unilateral nephrectomy, early in life is associated with an increased risk of kidney injury. Available data suggest that the risk of developing kidney injury is greater in children with an early acquired (EA-SFK) compared with congenital SFK.
- Hyperfiltration and reduced nitric oxide bioavailability were evident in both congenital and early acquired sheep models of SFK at eight months of age. The degree of hyperfiltration was greater in the model of EA-SFK, but only the congenital SFK animals displayed hypertension and albuminuria. This may reflect the different period of time since the kidney was lost in each model. Longer follow-up is required to determine if the enhanced hyperfiltration in the EA-SFK model will result in an accelerated decline in kidney function with aging.
- A better understanding of the underlying mechanisms could lead to the development of novel therapeutic interventions to attenuate glomerular hyperfiltration, protect the kidney, and improve future outcomes for children with an SFK.

## Data Availability

The data underlying this article will be shared on reasonable request to the corresponding author.

## Abbreviations

AA+D: amino acid and dopamine
ADMA: asymmetric dimethylarginine
BP: blood pressure
CKD: chronic kidney disease
C-SFK: congenital solitary functioning kidney
^51^Cr EDTA: ^51^Chromium ethylenediaminetetraacetic acid
EA-SFK: early acquired solitary functioning kidney
GFR: glomerular filtration rate
HR: heart rate
KIMONO: KIdney of MONofunctional Origin study
L-NAME: Nω-nitro-l-arginine methyl ester
MAP: mean arterial pressure
MARP: Monash Animal Research Platform
MRI: magnetic resonance imaging
NO: nitric oxide
NOS: nitric oxide synthase
NOx: total nitrate+nitrite
PAH: para-aminohippuric acid
PAS: Periodic acid–Schiff
PC-MRI: phase contrast magnetic resonance imaging
PRA: Plasma renin activity
RBF: renal blood flow
RFR: renal functional reserve
RVR: renal vascular resistance
SFK: solitary functioning kidney
i.v.: intravenous
unix: unilateral nephrectomy
